# Small Molecule Inhibitors of *Staphylococcus aureus* RnpA Alter Cellular mRNA Turnover, Exhibit Antimicrobial Activity, and Attenuate Pathogenesis

**DOI:** 10.1371/journal.ppat.1001287

**Published:** 2011-02-10

**Authors:** Patrick D. Olson, Lisa J. Kuechenmeister, Kelsi L. Anderson, Sonja Daily, Karen E. Beenken, Christelle M. Roux, Michelle L. Reniere, Tami L. Lewis, William J. Weiss, Mark Pulse, Phung Nguyen, Jerry W. Simecka, John M. Morrison, Khalid Sayood, Oluwatoyin A. Asojo, Mark S. Smeltzer, Eric P. Skaar, Paul M. Dunman

**Affiliations:** 1 Department of Pathology and Microbiology, University of Nebraska Medical Center, Omaha, Nebraska, United States of America; 2 Department of Microbiology and Immunology, University of Arkansas for Medical Sciences, Little Rock, Arkansas, United States of America; 3 Department of Microbiology and Immunology, Vanderbilt University Medical Center, Nashville, Tennessee, United States of America; 4 Department of Molecular Biology and Immunology, University of North Texas Health Science Center, Fort Worth, Texas, United States of America; 5 Department of Electrical Engineering, University of Nebraska, Lincoln, Nebraska, United States of America; 6 Department of Microbiology and Immunology, University of Rochester, Rochester, New York, United States of America; Dartmouth Medical School, United States of America

## Abstract

Methicillin-resistant *Staphylococcus aureus* is estimated to cause more U.S. deaths annually than HIV/AIDS. The emergence of hypervirulent and multidrug-resistant strains has further amplified public health concern and accentuated the need for new classes of antibiotics. RNA degradation is a required cellular process that could be exploited for novel antimicrobial drug development. However, such discovery efforts have been hindered because components of the Gram-positive RNA turnover machinery are incompletely defined. In the current study we found that the essential *S. aureus* protein, RnpA, catalyzes rRNA and mRNA digestion *in vitro*. Exploiting this activity, high through-put and secondary screening assays identified a small molecule inhibitor of RnpA-mediated *in vitro* RNA degradation. This agent was shown to limit cellular mRNA degradation and exhibited antimicrobial activity against predominant methicillin-resistant *S. aureus* (MRSA) lineages circulating throughout the U.S., vancomycin intermediate susceptible *S. aureus* (VISA), vancomycin resistant *S. aureus* (VRSA) and other Gram-positive bacterial pathogens with high RnpA amino acid conservation. We also found that this RnpA-inhibitor ameliorates disease in a systemic mouse infection model and has antimicrobial activity against biofilm-associated *S. aureus*. Taken together, these findings indicate that RnpA, either alone, as a component of the RNase P holoenzyme, and/or as a member of a more elaborate complex, may play a role in *S. aureus* RNA degradation and provide proof of principle for RNA catabolism-based antimicrobial therapy.

## Author Summary

The last decade has witnessed a mass downsizing in pharmaceutical antibiotic drug discovery initiatives. This has posed a major healthcare issue that will likely worsen with time; antibiotic resistant bacteria continue to emerge while advances in new therapeutic options languish. In the current body of work, we show that agents that limit bacterial RNA turnover have potential as a new class of antibiotics. More specifically, our findings indicate the essential bacterial protein, RnpA, exhibits *in vitro* ribonuclease activity and either alone and/or as a member of the RNase P holoenzyme, may contribute to the RNA degradation properties of *Staphylococcus aureus*, a predominant cause of hospital and community bacterial infections. Accordingly, using high throughput screening we identified small molecule inhibitors of RnpA's *in vitro* RNA degradation activity. One of these agents, RNPA1000, was shown to limit *S. aureus* mRNA turnover and growth. RNPA1000 also limited growth of other important Gram-positive bacterial pathogens, exhibited antimicrobial efficacy against biofilm associated *S. aureus* and protected against the *S. aureus* pathogenesis in an animal model of infection. When taken together, our results illustrate that components of the bacterial RNA degradation machinery have utility as antibiotic drug-discovery targets and that RNPA1000 may represent a progenitor of this new class of antibiotics.

## Introduction

*Staphylococcus aureus* infections are often associated with high rates of morbidity and mortality [1]. Indeed, reports estimate that in 2005 the organism caused more U.S. deaths than HIV/AIDS [2], [3]. The emergence of vancomycin-resistant and hypervirulent strains has further accentuated the need for novel anti-staphylococcal agents [4], [5]. Bacterial RNA processing and degradation are required cellular process that could be exploited for antibiotic drug discovery.

Much of our understanding of bacterial RNA degradation comes from studies of *Escherichia coli* where bulk mRNA decay is thought to be catalyzed by a holoenzyme complex (RNA degradosome), which consists of at least four subunits: RNase E (*rne*), RNA helicase (*rhlB*), enolase (*eno*), and PNPase (*pnpA*) [6]. RNase E is an essential ribonuclease and a key component of the degradosome complex; it serves as a scaffold for the assembly of other members of the RNA degradosome and catalyzes the initial endoribonucleolytic event during substrate degradation [7], [8]. Based on its essentiality, RNase E could be considered an appropriate target for antibiotic drug discovery. However, many Gram-positive bacteria, including *S. aureus*, lack a RNase E ortholog [9]. As a consequence, their degradation components and mechanism(s) of mRNA decay are less understood.

Recent studies suggest that at least two ribonucleases, RNase J1 and RNase Y, contribute to bulk mRNA degradation within *Bacillus subtilis*, and presumably other Gram-positive bacteria. *B. subtilis* ribonuclease J1 is a bifunctional ribonuclease, with 5′ exonuclease and endonuclease activities, that mediates mRNA degradation *in vitro* [10], [11]. The enzyme has also been found to interact with enolase (component of the *E. coli* RNA degradosome) and RNase J1 depleted *B. subtilis* strains demonstrate a moderate decrease in mRNA decay, suggesting that it may be the functional equivalent to *E. coli* RNase E [10], [12], [13]. However, mRNA turnover still occurs in RNase J1 diminished cells and RNA species containing 5′ strong-hairpin structures are not effectively degraded by the enzyme, indicating that additional factors are likely to contribute to *B. subtilis* cellular RNA degradation [14]. Ribonuclease Y is a recently identified endonuclease that can cleave mRNA molecules containing high-order secondary structures, globally affects cellular messenger RNA turnover and may ostensibly work in concert with RNase J1 to mediate bulk RNA decay [15]. Consistent with that possibility, recent two-hybridization studies revealed that RNase J1 and RNase Y are likely to interact with one another and with other proteins that are presumably members of the *B. subtilis* degradosome, including 6-phospho-fructokinase (Pfk), Enolase, PNPase, and the RNA helicase CshA [12], [16]. Both RNase J1 and RNase Y are essential enzymes and, in that regard, could be considered targets for antimicrobial drug discovery [17]. However, it remains to be seen whether RNase J1, RNase Y, and/or previously uncharacterized ribonucleases modulate mRNA decay within *S. aureus*.

In the current body of work we set out to empirically identify *S. aureus* RNA degradation factors, with the expectation that they would represent promising antimicrobial drug development targets. To do so, we exploited the fact that *S. aureus* owes its ability to cause infection, in part, to the temporal expression of an expansive repertoire of virulence factors, many of which are regulated in a cell density-dependent manner during laboratory culture conditions [18]. This, combined with recent reports indicating that bacterial pathogens, including *S. aureus*, govern gene expression by modulating the mRNA turnover of target transcripts [19], [20], [21] led to the prediction that growth phase regulated changes in *S. aureus* virulence factor expression occur at the level of mRNA degradation and that the proteins involved in this process may include members of the organism's RNA degradation machinery. Accordingly, Affymetrix GeneChips were used to compare the mRNA decay rates of well-characterized *S. aureus* virulence factors during exponential- and stationary- phase growth.

Results revealed that the mRNA turnover properties of many *S. aureus* virulence factor transcripts differed between the two growth phases. Furthermore, and of direct relevance to the current work, the global mRNA decay properties of exponential and stationary phase cells were found to be dramatically different; 884 *S. aureus* mRNA species were stabilized during stationary phase growth. Among the genes whose expression correlated with mRNA decay was the protein component of ribonuclease P (RNase P), RnpA, suggesting that it may play a role in bulk mRNA turnover. Consistent with that possibility, we show that recombinant *S. aureus* RnpA exhibits ribonuclease activity *in vitro* and RnpA depleted cells exhibit reduced mRNA degradation, indicating that RnpA-alone, RNase P, or RnpA in complex with other cellular factors contributes to *S. aureus* mRNA degradation. Because RnpA is an essential *S. aureus* enzyme with low amino acid conservation with mammalian proteins, we hypothesized that it may be an appropriate target for antimicrobial drug discovery. Accordingly, high through-put and secondary screening assays were used to identify small molecule inhibitors of RnpA-mediated *in vitro* RNA degradation. One of these agents was shown to inhibit *S. aureus* cellular mRNA turnover, exhibited antimicrobial activity against MRSA, VISA and VRSA, as well as other Gram-positive pathogens with high RnpA conservation, and limited pathogenesis in a murine acute lethal model of infection. Collectively these results suggest that RnpA alone, or as a member of a more elaborate complex, may contribute to the mRNA degradation properties of *S. aureus* and validate its utility as an antimicrobial drug discovery target.

## Results

Many *S. aureus* virulence factors are expressed in a cell density dependent manner during growth in laboratory culture conditions. In general, cell surface virulence determinants are predominantly expressed during exponential phase growth, whereas secreted virulence factors are primarily expressed during stationary phase growth [18]. Recent studies indicate that regulated changes in *S. aureus* mRNA turnover, in part, effect the expression of the organism's virulence determinants [22]. Accordingly, we predicted that alterations between the mRNA turnover properties of exponential and stationary phase cells may differ in a manner that correlate with changes in virulence factor expression.

### Growth-phase dependent alterations in *S. aureus* mRNA turnover

**Figure 1:**
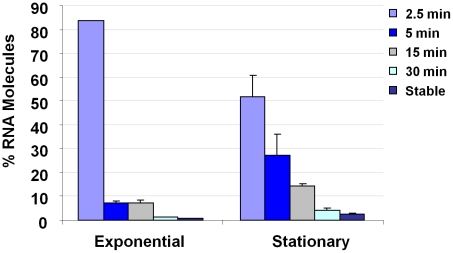
*S. aureus* growth phase mRNA turnover measurements. Plotted are the percent of detectible mRNA species (Y-axis) with a half life of ≤2.5, 5, 15, 30, or >30 min during exponential- and/or stationary- phase growth (X-axis).

Affymetrix GeneChips were used to compare the mRNA turnover properties of exponential- and stationary- phase *S. aureus* cells. To do so, *S. aureus* strain UAMS-1 was cultured to either mid-exponential or stationary phase growth. *De novo* transcript synthesis was arrested by the addition of rifampin and aliquots were removed at various post-transcriptional arrest time points. The RNA from these samples was labeled, applied to Affymetrix GeneChips, and the half-life of each mRNA species was determined, as previously described [19], [20]. As predicted, results revealed that the mRNA turnover properties of many (41%) virulence factor transcripts differed between the two growth phases, suggesting that regulated changes in mRNA turnover may affect their expression (Supplementary Table S1.). Moreover, it was observed that the organism produced at least five stationary phase specific small stable RNAs (SSRs), a hypothesized class of regulatory non-coding RNA molecules (Supplementary Table S2.; [19], [20]). Further, the global mRNA turnover properties of exponential- and stationary-phase cells differed considerably. Consistent with previous measurements, it was found that most (90%) exponential phase transcripts are rapidly degraded (half life of ≤5 min), 9% exhibit intermediate stability (half life of >5 min but ≤30 min), and 1% are stable (half life of ≥30 min) [19], [20]. However, during stationary phase growth, 76%, 21%, and 3% of mRNA species exhibit short, intermediate, and stable half lives, respectively (Figure 1). We anticipated that the observed stationary phase-dependent stabilization of many mRNA species would reflect diminished expression of a member(s) of the *S. aureus* RNA decay machinery; neither RNase J1 or RNase Y were found to be differentially expressed in a growth phase dependent manner. Among the 367 genes repressed during stationary phase growth was *rnpA*, which codes for the protein component of ribonuclease P (RNase P; Supplementary Table S1).

### *S. aureus* RnpA exhibits ribonuclease activity

RNase P is an ubiquitous enzyme that catalyzes maturation of the 5′ end of precursor tRNAs [23], [24], [25]. The enzyme is unique by virtue of the fact that it is a ribonucleoprotein complex, which includes a single ribozyme RNA molecule and at least one protein component. Within bacteria both the ribozyme (*rnpB*) and protein (RnpA) components are required for cell viability; *rnpB* mediates tRNA processing *in vitro*, whereas RnpA facilitates *rnpB*/substrate binding at physiologically relevant magnesium concentrations [26], [27], [28], [29]. In addition to catalyzing tRNA maturation, *E. coli* and *B. subtilis* RNase P have been found to digest certain double-stranded RNA templates, such as guide-RNAs and 4.5s RNA [30]. Cleavage of those templates strictly requires RnpA [31], [32], raising the possibility that RNase P mediated RNA digestion may be dependent on *rnpB*, RnpA, or both. Domain searches (http://smart.embl.de/) [33], [34] revealed that *S. aureus* RnpA residues 40–111 best conform to a ribonuclease-like motif (data not shown) and several RNA binding sites are embedded within this region [35]. Taking these observations into consideration, we predicted that RnpA, either alone or, more likely, as a component of RNase P holoenzyme or a more elaborate complex, affects *S. aureus* RNA degradation.

**Figure 2:**
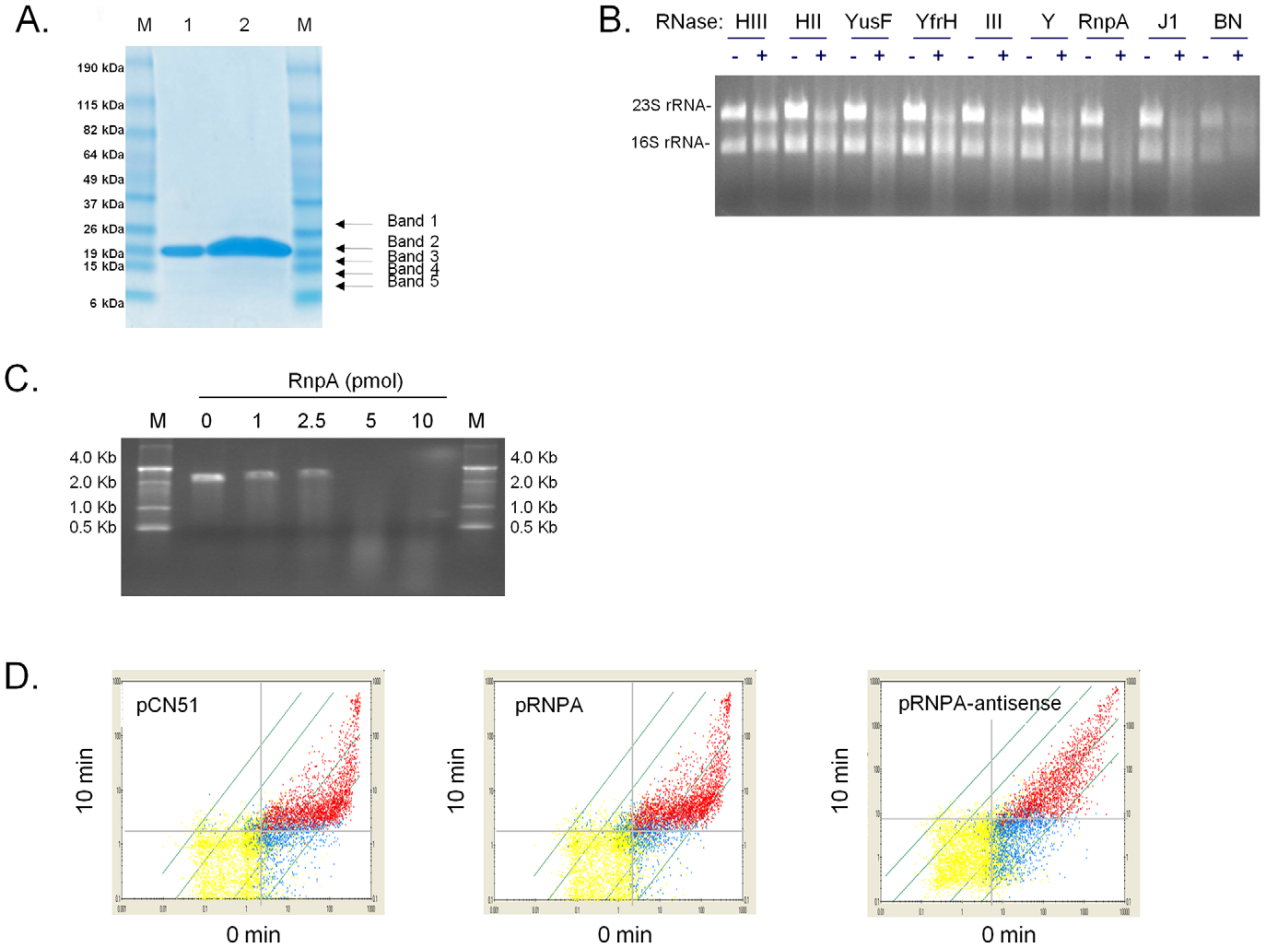
*S. aureus* RnpA catalyzes rRNA and mRNA digestion. (**A**) SDS-PAGE of purified recombinant *S. aureus* RnpA; shown are molecular markers (Lanes M), 2.5 µg and 25 µg elution products (Lanes 1 and 2, respectively). The band at ∼17.2 kDa (solid arrow; Band 2) was confirmed to be *S. aureus* RnpA by tandem mass spectrometry (Wistar Institute; Philadelphia, PA), whereas top-hits for minor contaminants (dashed arrows) were determined to be *E. coli* 50S ribosomal protein L3 (Band 1) or *S. aureus* RnpA polypeptide fragments, corresponding to amino acids 11–107 (Bands 3 and 4) or 12–107 (Band 5). (**B**) Gel-mobility of 1 µg of total *S. aureus* RNA following 60 min incubation in the absence (−) or presence (+) of 50 pmol of each putative ribonuclease (indicated) in 1X reaction buffer (2 mM NaCl, 2 mM MgCl_2_, 50 mM Tris-HCl, pH 6.0). Recombinant *S. aureus* RnpA catalyzed RNA digestion; equivalent amounts of other putative *S. aureus* ribonucleases demonstrated little or no ribonuclease activity. (**C**) Mobility of 0.5 pmol *in vitro* transcribed *spa* mRNA following 60 min incubation in the absence (0 pmol) or presence of the indicated amount of RnpA protein in 1X reaction buffer. Recombinant *S. aureus* RnpA catalyzed digestion of *spa* mRNA; molecular weight markers (M) are shown. (**D**) Plotted are measurements for all mRNA species measured on a GeneChip at 0 (X-axis) and 10 min (Y-axis) post-transcriptional arrest. Red data points represent all transcripts that are considered present by Affymetrix algorithms at both 0 and 10 min following transcriptional arrest. Blue data points represent transcripts that were considered present in one sample but not detectable in the second. Yellow data points represent transcripts that are not expressed/detectible in either RNA sample. Comparisons of the transcript titer of a given data point at 0 min to that of 10 min post-transcriptional arrest serves as a measurement of the degradation of that particular mRNA species. Grey dashed line indicates the lower limit of sensitivity for each sample. A total of 88% and 87% of all transcripts produced in cells harboring vector (pCN51; left panel) or overexpressing RnpA (pRNPA; middle panel), respectively, exhibit a decrease in transcript titer of ≥2-fold following 10 min transcriptional arrest. Conversely, many RNA species are stabilized in RnpA depleted cells (pRNPA-A.S.; right panel). Western blotting confirmed the amount of *S. aureus* RnpA protein present in each strain (Supplemental Figure S1B.).

Consistent with those possibilities, recombinant *S. aureus* RnpA was found to catalyze digestion of rRNA and staphylococcal protein A (*spa*) mRNA (Figures 2B and 2C), as well as three other mRNA species tested (data not shown). Other putative *S. aureus* ribonucleases including RNase III, RNase HII, RNase HIII, RNase Y, RNase J1, and BN did not exhibit equivalent RNA degradation activity during these assay conditions (Figure 2B). SDS-PAGE and matrix-assisted laser desorption/ionization (MALDI) analysis confirmed that the observed ribonuclease activity was associated with the presence of *S. aureus* RnpA (Figure 2A). None the less, SDS-PAGE assessment of approximately 1000-fold excess (25 µg) of RnpA purification product used in the aforementioned ribonuclease assays revealed trace amounts of four additional polypeptides within the protein preparation, raising the possibility that contaminating *E. coli* ribonucleases may be present with our RnpA product. MALDI analysis revealed the identity of these proteins to be *E. coli* ribosomal protein L3, and three *S. aureus* RnpA fragments, presumably reflecting proteolytic degradation of full length RnpA during protein preparation as opposed to mature alternative translation products. Importantly, no *E. coli* ribonucleases were detected, suggesting that the protein preparation's ribonucleolytic activity could be attributed to *S. aureus* RnpA. Moreover, reverse transcriptase mediated PCR revealed that *E. coli rnpB* was undetectable within our preparation, establishing that RnpA ribonuclease activity was not due to the formation of chimeric RNase P molecules consisting of *S. aureus* RnpA and *E. coli rnpB* RNA. Indeed, *in vitro* synthesized *E. coli rnpB* neither catalyzed *S. aureus* RNA degradation (alone) nor affected the activity of RnpA-mediated RNA digestion during both standard and elevated Mg^+2^ reaction conditions (data not shown).

### *S. aureus* RnpA is an essential enzyme that affects cellular mRNA degradation

Small molecule inhibitors of essential bacterial RNA turnover proteins are expected to interfere with bacterial growth and represent a new class of antimicrobial agents. In that regard, *S. aureus* RnpA is a reported essential enzyme [36], [37] and thus could be considered a target for chemotherapeutic development. Indeed, induction of an antisense RNA molecule that is predicted to be complementary to the −34 to +353 *rnpA* mRNA translation start site (under control of the cadmium chloride inducible promoter of plasmid, pCN51 [38]) limited *S. aureus* proliferation in the presence of 10 µM inducer. Conversely, no growth defects were observed for cells expressing the corresponding sense strand RNA molecule or the antisense plasmid strain in the absence of inducer (Supplemental Figure S1; data not shown). These results support the work of others and indicate that *S. aureus* RnpA is an essential protein. Further, using this *rnpA* antisense RNA system we assessed whether RnpA affects *S. aureus* cellular mRNA turnover. Accordingly, the RNA degradation properties where measured for cells harboring plasmid vector alone or cells containing plasmid borne copies of *rnpA* mRNA or *rnpA* antisense RNA during growth in the presence of 2.5 µM CaCl_2_. As shown in Supplementary Figures S1A and S1B, 2.5 µM cadmium chloride was empirically determined to be the optimal concentration that allowed increased- or decreased- RnpA production within *rnpA* mRNA or *rnpA* antisense expressing strains, respectively, but did not limit bacterial growth of the antisense RNA producing strain. Accordingly, RNA turnover analyses revealed that diminished RnpA levels correlated with the stabilization of many mRNA species, suggesting that the enzyme either alone, as a member of the RNase P holoenzyme, and/or as another RnpA-complex contributes to bulk cellular RNA degradation (Figure 2D). More specifically, it was found that 88% and 87% of all exponential phase transcripts produced in RnpA overexpressing and vector containing cells exhibited a half life of less than 10 min, respectively. Conversely, 63% of transcripts produced in RnpA depleted cells exhibited a half life of less than 10 min, suggesting that the protein contributes to *S. aureus* mRNA turnover (Figure 2D). The finding that RnpA overexpression did not accelerate cellular RNA degradation suggests that either the protein did not reach a concentration that effectively increases RNA turnover or that the protein's RNA degradation activity is dependent on co-factors, which remain at wild type levels under these experimental conditions.

### Identification of small molecule inhibitors of RnpA-mediated RNA degradation

**Figure 3:**
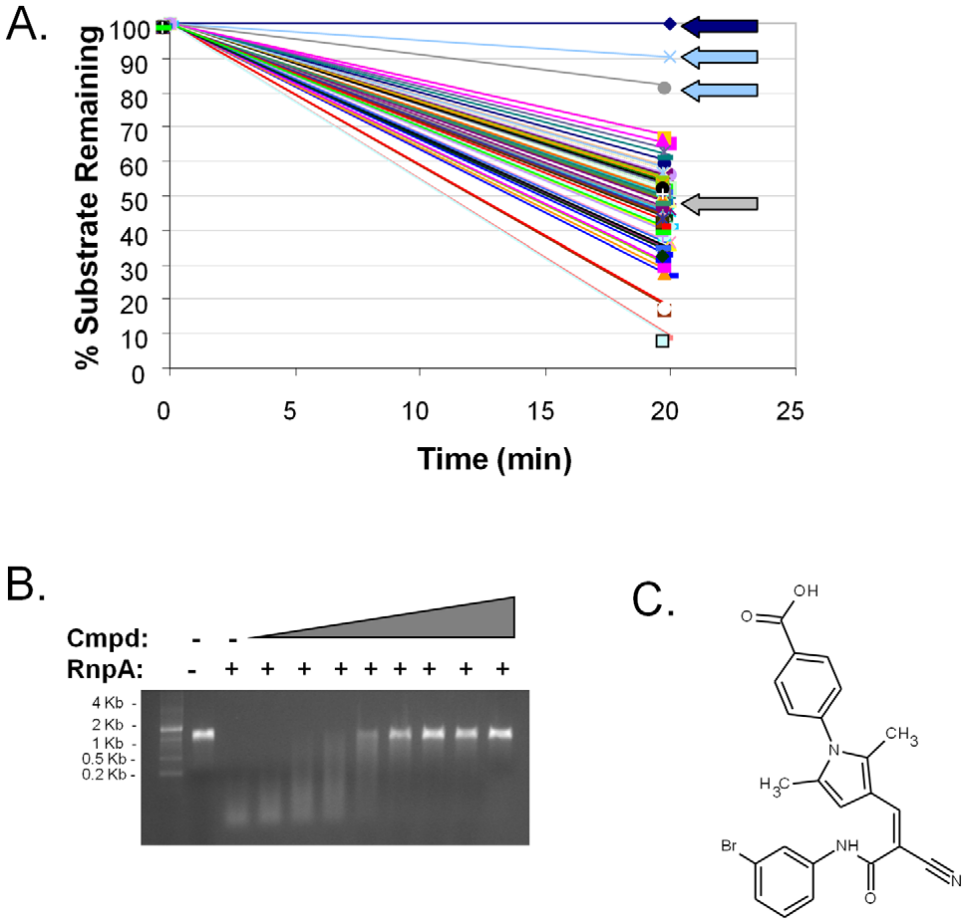
Identification of small molecule inhibitors of RnpA-mediated RNA degradation. (**A**) Representative screening effort results; dark blue arrow indicates substrate alone (negative control); grey arrow indicates enzyme (positive control); light-blue arrows indicate compounds that inhibited RnpA activity by ≥50%. (**B**) An agarose gel-based assay was used to distinguish *bona-fide* RnpA inhibitors from primary screening artifacts. Shown is the gel mobility of molecular weight marker, *spa* mRNA in the absence (−) or presence (+) of 20 pmol RnpA and RnpA-mediated *spa* mRNA degradation in the presence of increasing concentrations of RNPA1000, as described in Materials and Methods. (**C**) Structure of RnpA-inhibitory molecule RNPA1000.

The above results indicate that *S. aureus* RnpA is an essential enzyme that exhibits *in vitro* ribonuclease activity and either alone, as a component of RNase P or in concert with other cellular components participates in bulk RNA degradation. Moreover, the protein is well conserved across Gram-positive bacteria but lacks amino acid conservation with mammalian proteins, making it an attractive target for novel antibiotic drug development. Accordingly, we set out to exploit the protein's *in vitro* ribonuclease activity as a means to identify RnpA inhibitory agents; a fluorescence-based high through-put assay was used to screen 29,066 commercial compounds (ActiProbe-25K and Natural product libraries; Timtec; Newark, DE) for small molecule inhibitors of RnpA-mediated *in vitro* RNA degradation (Figure 3A). In total, fourteen molecules inhibited the enzyme's RNA turnover activity by ≥50%. A gel-based secondary assay confirmed that five of these molecules were *bona-fide* inhibitors of RnpA-mediated RNA degradation (Figure 3B). One of these compounds, RNPA1000 (Figure 3C; IC_50_  = 100–125 µM), did not affect the activity of the commercially available *E. coli* RNase HI, RNase A, RNase I or in-house purified *S. aureus* RNase J1 at any concentration tested (0–750 µM), but did mildly inhibit *E. coli* RNase III activity (IC_50_  = 500–750 µM; data not shown). These and other data (see below) suggest that RNPA1000 may have specificity for *S. aureus* RnpA, yet as with any small molecule we cannot rule out the possibility that the agent may also affect other *S. aureus* enzymes. To assess whether RnpA-inhibitory agents exhibit potential as antimicrobials, a series of experiments were performed to evaluate whether RNPA1000 inhibited *S. aureus* growth and could limit *S. aureus* pathogenesis in a systemic model of infection.

### *In vitro* antimicrobial activity of RnpA-inhibitor RNPA1000

As shown in Table 1, RNPA1000 demonstrated moderate antimicrobial activity against two well-characterized genotypically diverse *S. aureus* isolates, UAMS-1 (clinical osteomyelitis isolate; MIC 26 µg/ml) and USA300–0114 [predominant cause of U.S. community-associated methicillin resistant *S. aureus* infections (MRSA); MIC 23 µg/ml], as well as representatives of other major MRSA lineages circulating throughout the U.S. [39]. Likewise, RNPA1000 demonstrated antimicrobial activity against vancomycin-intermediate susceptible *S. aureus* (VISA) and vancomycin resistant *S. aureus* (VRSA). Time kill assays revealed that RNPA1000 acts as a bacteriostatic agent (Supplemental Figure S2A), and that it does not affect the antimicrobial activities of other anti-staphylococcal agents, including vancomycin, daptomycin, or rifampicin (data not shown), but does mildly increase the potency of oxacillin (Supplemental Figure S2B and S2C). The RnpA-inhibitor also exhibited antimicrobial activity against *Staphylococcus epidermidis*, antibiotic susceptible and multi-drug resistant *Streptococcus pneumoniae*, *Streptococcus pyogenes*, *Streptococcus agalactiae*, and *Bacillus cereus*. RNPA1000 also showed mild activity against *Enterococcus faecalis*, *Enterococcus faecium* and vancomycin resistant *E. faecium* (VRE), but did not affect *Escherichia coli* or *Acinetobacter baumannii* growth (Table 1). The latter was expected because *E. coli* and *A. baumannii* RnpA share limited amino acid identity (24% and 26%, respectively) with *S. aureus* RnpA (Supplemental Figure S3). Moreover, purified *A. baumannii* RnpA did not demonstrate ribonuclolytic activity in our *in vitro* assay conditions (data not shown). Enterococci susceptibility to RNPA1000 was increased from an MIC of 64 µg/ml to 32 µg/ml in the presence of the efflux pump inhibitor reserpine, suggesting that enterococci may be inherently susceptible to the RnpA inhibitor. Conversely, the efflux inhibitor had no effect on *A. baumannii* RNPA1000 susceptibility (Table 1). Taken together, these results indicate that bacterial RNPA1000 susceptibility correlates with amino acid similarity to *S. aureus* RnpA and the enzyme's *in vitro* RNA degradation activity.

**Table 1:** *In vitro* RNPA1000 antimicrobial properties.

Organism (Phenotype) a	Strain b	MIC (µg/ml) c	Organism (Phenotype) a	Strain b	MIC (µg/ml) c
*S. aureus* (MRSA)	USA100	26	*S. pneumoniae* (MDR)	Isolate 4	32
*S. aureus* (MRSA)	USA200	32	*S. pneumoniae* (MDR)	Isolate 5	16
*S. aureus* (MRSA)	USA300	23			
*S. aureus* (MRSA)	USA400	23	*S. pyogenes*	Isolate 1	8
*S. aureus* (MRSA)	USA500	23	*S. sanguis*	Isolate 1	16
*S. aureus* (MRSA)	USA600	32	*S. bovis*	49147	32
*S. aureus* (MRSA)	USA700	32			
*S. aureus* (MRSA)	USA800	23	*E. faecalis*	Isolate 1	64
*S. aureus* (MRSA)	USA900	32	*E. faecalis*	Isolate 2	64
*S. aureus* (MRSA)	USA1000	29	*E. faecalis*	Isolate 3	64
*S. aureus* (MRSA)	USA1100	32	*E. faecalis*	Isolate 4	64
*S. aureus* (MSSA)	UAMS-1	26	*E. faecalis*	Isolate 5	64
*S. aureus* (VISA)	NRS1	32	*E. faecium*	Isolate 1	64
*S. aureus* (VISA)	NRS3	16	*E. faecium*	Isolate 2	64
*S. aureus* (VISA)	Isolate 3	16	*E. faecium*	Isolate 3	64
*S. aureus* (VISA)	Isolate 4	32	*E. faecium*	Isolate 4	64
*S. aureus* (VISA)	Isolate 5	16	*E. faecium*	Isolate 5	64
			+ reserpine	Isolate 5	32
*S. aureus* (VRSA)	VRS1	16			
*S. aureus* (VRSA)	VRS10	32	*E. faecium* (VRE)	Isolate 1	64
			*E. faecium* (VRE)	Isolate 2	64
*S. epidermidis*	Isolate 1	16	*E. faecium* (VRE)	Isolate 3	32
*S. epidermidis*	Isolate 2	8	*E. faecium* (VRE)	Isolate 4	64
*S. epidermidis*	Isolate 3	8	*E. faecium* (VRE)	Isolate 5	32
*S. epidermidis*	Isolate 4	8			
*S. epidermidis*	Isolate 5	8	*B. cereus*	Isolate 1	8
*S. agalactiae*	Isolate 1	16	*E. coli*	Isolate 1	>64
*S. agalactiae*	Isolate 2	32	*E. coli*	Isolate 2	>64
*S. agalactiae*	Isolate 3	32	*E. coli*	Isolate 3	>64
*S. agalactiae*	Isolate 4	32	*E. coli*	Isolate 4	>64
			*E. coli*	Isolate 5	>64
*S. pneumoniae*	Isolate 1	16			
*S. pneumoniae*	Isolate 2	16	*A. baumannii*	Isolate 1	>64
*S. pneumoniae*	Isolate 3	16	*A. baumannii*	Isolate 2	>64
*S. pneumoniae*	Isolate 4	32	*A. baumannii*	Isolate 3	>64
*S. pneumoniae*	Isolate 5	16	*A. baumannii*	Isolate 4	>64
			*A. baumannii*	Isolate 5	>64
*S. pneumoniae* (MDR)	Isolate 1	32	+ reserpine	Isolate 5	>64
*S. pneumoniae* (MDR)	Isolate 2	32			
*S. pneumoniae* (MDR)	Isolate 3	16			

a Organism and relevant antibiotic resistance phenotype (in parentheses); methicillin resistant *S. aureus* (MRSA); vancomycin intermediate *S. aureus* (VISA); vancomycin resistant *S. aureus* (VRSA); multidrug resistant *S. pneumoniae* (MDR); vancomycin resistant *E. faecium* (VRE).

b With the exception of *S. aureus* and *S. bovis*, all strains were clinical blood isolates; USA-types (U.S. MRSA lineages) were obtained from the Centers for Disease Control and Prevention; *S. aureus* strain UAMS-1 is a clinical osteomyelitis isolate; isolates NRS1, NRS3, VRS1 and VRS10 were obtained through the Network on Antimicrobial Resistance in *Staphylococcus aureus* (NARSA); *S. bovis* strain ATCC49147 was obtained from the American Type Culture Collection (ATCC).

c Minimal inhibitory concentration (MIC) was determined following the Clinical and Laboratory Standards Institute (CLSI) guidelines for antimicrobial susceptibility testing. Each *S. aureus* strain was tested 5 times; other organisms were tested in duplicate. *S. aureus* isolates were subsequently more accurately measured as described within Materials and Methods.

### *In vivo* antimicrobial efficacy of RnpA-inhibitor RNPA1000

**Figure 4:**
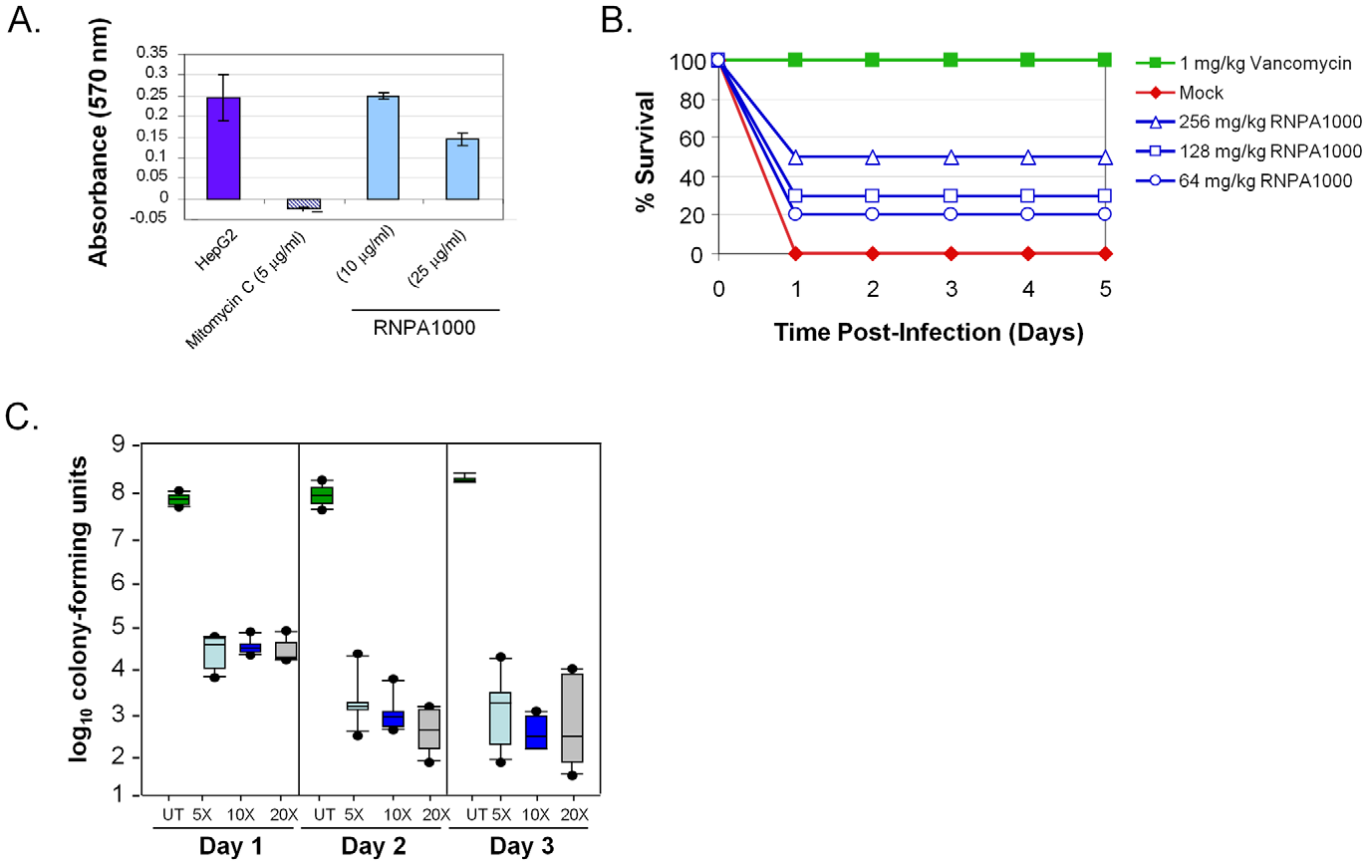
RnpA-inhibitor characterization. (**A**) MTT-cytotoxicity assay results of HepG2 cells exposed to compound solvent (DMSO; negative control), Mitomycin C (positive control), and indicated amount of RNPA1000. (**B**) Shown are the average daily (X-axis) percent surviving animals (Y-axis) following no treatment (closed diamonds; negative control), vancomycin treatment (closed squares; 1 mg/kg; positive control), or RNPA1000 treatment; 64 mg/kg (open circles); 128 mg/kg (open squares), 256 mg/kg (open triangles). Experiment was repeated twice; replicate measurements are shown in Supplemental Table S3. (**C**) *In vitro* biofilm assay results; graphed are the number of catheter-associated *S. aureus* following 1, 2, or 3 days of no antimicrobial treatment (untreated; UT) or exposure to 5, 10, or 20 times the MIC for RNPA1000. Boxes define the interval between the 25^th^ and 75^th^ percentile. Bars extending upward indicate the boundary defined by the value 1.5X higher than the 75^th^ percentile while those extending downward indicate the boundary defined by a value 1.5X lower than the 25^th^ percentile. Filled circles indicate individual values outside these two extremes.

Next we assessed whether RnpA-inhibitory agent concentrations corresponding to the effective bacterial MIC values (10–50 µg/ml) elicited human cell cytotoxicity. MTT cell proliferation assay measurements revealed that 24 hr RnpA-inhibitor exposure did not cause human HepG2 cell toxicity at any concentration tested (data not shown). However, extended RNP1000 exposure (48 hr) elicited mild cytotoxicity at 25 µg/ml, which corresponds to the minimum inhibitory concentration of most MRSA lineages (Figure 4A), whereas higher concentrations exhibited increased toxicity (data not shown).

Clearly, the observed toxicity associated with RNPA1000 precludes its direct utility as an antimicrobial agent. None the less, we hypothesized that because RNPA1000 was not toxic during short- and only mildly toxic during extended- HepG2 exposure, it could serve as an appropriate tool to assess whether RnpA-inhibitory molecules are efficacious in a systemic mouse infection model. As shown in Figure 4B, subcutaneous injection of RNPA1000 limited the lethal effects of wild type *S. aureus* injected (4.55×10^5^ cfu/animal) into the intraperitoneal cavity of CD-1 mice. Although this bacterial inoculum (equivalent to 10–100 LD_50_s) resulted in 100% death of non-treated control animals within 24 hr, RNPA1000 provided protection in a dose-dependent manner. Administration of the highest RnpA-inhibitor dose (256 mg/kg) reproducibly resulted in 50% survival, whereas 128 mg/kg and 64 mg/kg resulted in 30% and 20% survival, respectively, over the course of study (Figure 4B; Supplemental Table S3). Notably, dosing regimens of compound (alone) did not affect animal survival at any of the concentrations tested (32 mg/kg, 64 mg/kg, 128 mg/kg, 256 mg/kg; Supplemental Table S3). Taken together, these results suggest that RNPA1000 limits bacterial pathogenicity within the acute lethal model of *S. aureus* infection with a median effective dose (ED_50_) between 64–256 mg/kg. While the combination of mild toxicity and high effective dose would exclude the compound from consideration as a therapeutic agent, RNPA1000 could be considered a platform for medicinal chemistry-based generation of more potent derivatives. More importantly, these results provide proof of concept that RnpA inhibitory agents are efficacious in a systemic mouse infection model and that RNPA1000 represents a tool to study the contribution of RnpA to infection processes.

### Antimicrobial efficacy of RnpA-inhibitor on biofilm-associated bacteria

The success of *S. aureus* as a bacterial pathogen can be attributable, in part, to its ability to form biofilms on implanted medical devices, which presumably provides a focus for bacterial dissemination to secondary host sites. One of the complicating issues in treating biofilm-associated infections is that biofilm-associated bacteria are inherently recalcitrant to antibiotic treatment. For instance, one recent *in vitro* study showed that despite using a strain that was intrinsically susceptible to each antibiotic, 5X MIC of daptomycin, linezolid, or vancomycin only reduced biofilm-associated bacteria by <2 logs following 24 hr treatment and none of these antibiotics cleared biofilm-associated *S. aureus* even when administered at 20X MIC over a course of 3 days [40]. Transcription profiling studies have revealed that despite being physiologically unique, biofilm-associated *S. aureus* resemble planktonic stationary phase cells [41]. Indeed, similar to stationary phase bacteria, *rnpA* expression is diminished 4.3 and 6.2- fold in *S. aureus* biofilm-associated and biofilm-detached bacteria, respectively, in comparison to exponential phase cells (Dunman and Horswill, unpublished). Because low levels of RnpA are likely to be present within biofilm-associated bacteria, we hypothesized that fewer RnpA-inhibitory molecules would be required to interfere with the protein's function and, consequently, antimicrobial activity. Thus, biofilm-associated *S. aureus* may exhibit considerable susceptibility to an RnpA-inhibitor, such as RNPA1000.

As shown in Figure 4C, treatment of biofilm-associated *S. aureus* with 5X MIC RNPA1000 for 24 hr resulted in a 3-log decrease in bacterial burden, suggesting that during short term exposure the agent is equally, if not more potent, than daptomycin, vancomycin, or linezolid. Further, while bacterial clearance was never achieved, increasing the length of exposure or RNPA1000 concentration enhanced antimicrobial activity. Maximal RNPA1000 antimicrobial potency (5-log reduction in biofilm-associated bacteria) compared favorably with the activities of commercially available antibiotics assessed in the same model and conditions (6-log decrease daptomycin, 5-log decrease linezolid; 4-log decrease vancomycin) [40]. Taken together, these results suggest that RnpA plays an important biological role in *S. aureus* biofilm maintenance, and that corresponding inhibitors may have expanded therapeutic utility in treating biofilm-associated infections.

### RNPA1000 affects *S. aureus* mRNA decay

**Figure 5:**
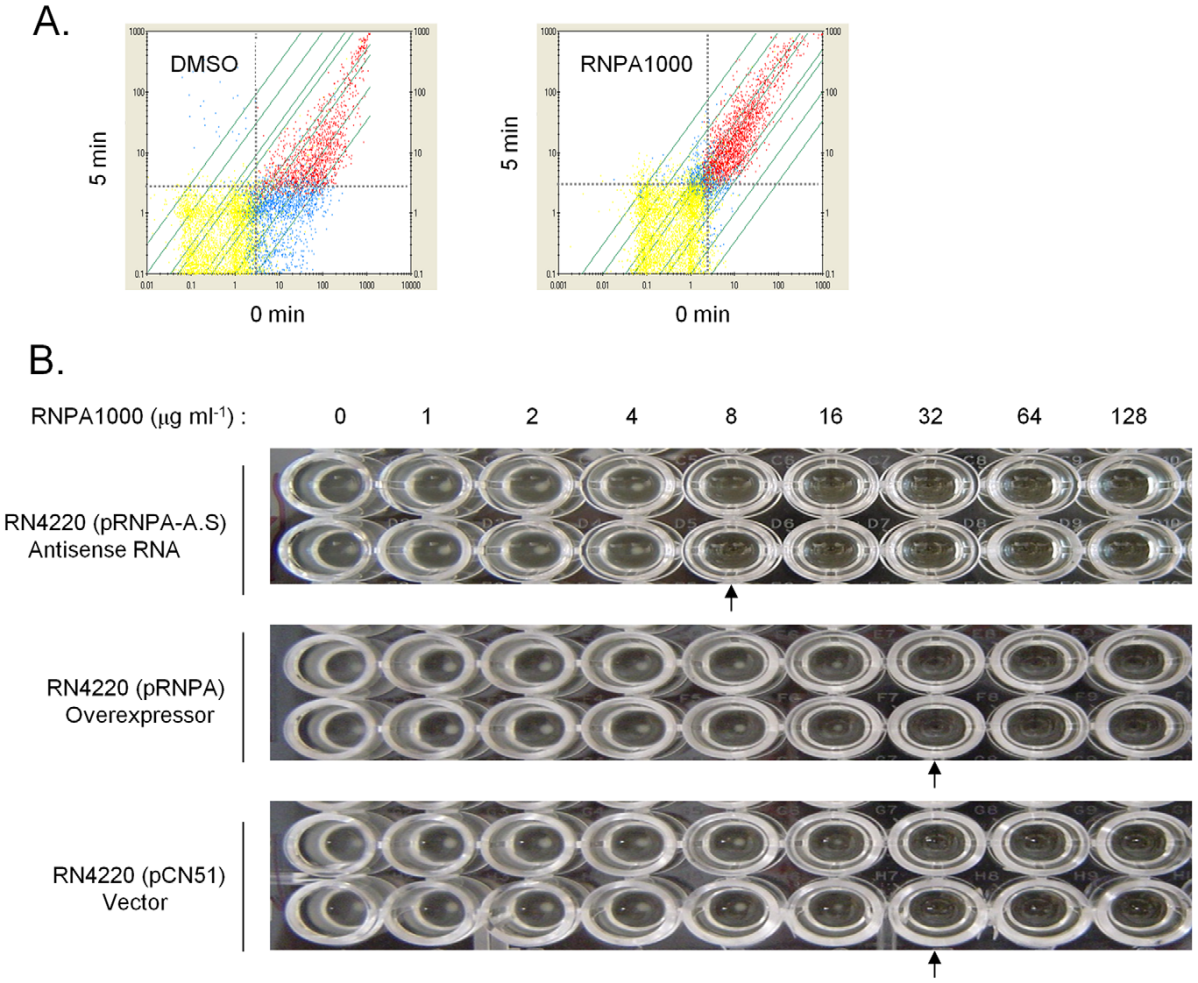
RNPA1000 characterization. Plotted are all GeneChip detected transcript levels at 0 and 5 min post-transcriptional arrest. Red, blue, and yellow data points represent all GeneChip measurable transcripts that were considered present, present in one sample but absent in second, or absent in both RNA samples, respectively. Grey dashed line indicates the lower limit of sensitivity for each sample. (**A**) A comparison of the mRNA levels of DMSO treated cells; 90% of all transcripts decrease ≥2-fold at 5 min post-transcriptional arrest (Left Panel). A comparison of the mRNA levels of cells challenged with sub-inhibitory concentration of RnpA-inhibitor; addition of the agent resulted in pronounced stabilization of *S. aureus* transcripts (Right Panel). (**B**) Microtiter plate assay illustrating the *in vitro* antimicrobial effects of indicated concentration of RNPA1000 (across top) against *S. aureus* RN4220 pRNPA-A.S. (RnpA depleted cells; top panel), RN4220 pRNPA (RnpA overexpressor cells; center panel) and RN4220 pCN51 (vector; bottom panel) when grown in the presence of 2.5 µM CdCl_2_. All strains were assessed twice; arrows indicate MIC values.

To assess whether the susceptibility of *S. aureus* to RNPA1000 was attributable to the inhibition of cellular RnpA, we initially attempted to isolate and characterize spontaneous mutants with reduced compound susceptibility. Attempts to isolate mutants that were resistant to 64 µg/ml RNPA1000 (2X MIC) were unsuccessful. By reducing the stringency it was found that the spontaneous RNPA1000 resistance frequency to 32 µg/ml RNPA1000 is 3.7×10^−13^, but further characterization of these mutants revealed that the resistance phenotype was lost following serial passage. As a second approach, we directly measured the mRNA turnover properties of *S. aureus* that were challenged with a sub-inhibitory concentration of RnpA-inhibitor (0.5X MIC). Following 30 min treatment, RNPA1000 reduced the mRNA degradation rate of *S. aureus* cells, in comparison to mock treated cells (Figure 5A). Thus, RnpA-inhibitory compounds reduce cellular mRNA degradation, presumably by limiting the enzyme's cellular function. While we cannot rule out the possibility that the agent affects other enzymes or, in particular other *S. aureus* ribonucleases, the mRNA turnover properties of RNPA1000 treated cells resembled that of RnpA depleted cells (Figure 2D), suggesting that the agent may be affecting the enzyme. To more directly determine whether RNPA1000's antimicrobial effects are mediated through cellular inhibition of RnpA we assessed the RNPA1000 susceptibility of *S. aureus* RnpA over- and under- producing cells. *S. aureus* harboring vector, or a plasmid copy of wild type *rnpA* mRNA or *rnpA* antisense RNA under control of the CdCl_2_ inducible promoter were grown in the presence of 2.5 µM inducer and increasing concentrations of RNPA1000. As stated above, this concentration of cadmium chloride induces mild changes in RnpA protein expression (RnpA overproduction or underproduction) but is modest enough that cellular growth is not affected. As shown in Figure 5B, both vector containing- and RnpA overproducing- cells exhibited an MIC of 32 µg ml^−1^, whereas the MIC of RnpA underproducing cells was 8 µg ml^−1^. The latter indicates that *S. aureus*' RNPA1000 susceptibility correlates to cellular RnpA levels and that the agent's antimicrobial mode-of-action is, in part, RnpA dependent. It is currently unclear why RnpA overexpression did not result in increased RNPA1000 tolerance; presumably enzyme levels did not reach concentrations needed to limit RNPA1000 effectiveness.

## Discussion

The emergence of community-acquired methicillin-resistant *S. aureus* (CA-MRSA) strains capable of causing serious infections in otherwise healthy individuals make the organism a more formidable pathogen now than perhaps any time since the beginning of the antibiotic era. Indeed, it is estimated that in 2005 alone the number of invasive MRSA infections in the U.S. reached approximately 94,360, with 18,650 resulting in fatal outcome [3]. Based on these statistics, *S. aureus* has passed acquired-immunodeficiency syndrome (AIDS) as a cause of death in the United States, further accentuating the need for new anti-staphylococcal agents.

The success of *S. aureus* as a human pathogen can, in part, be attributed to its ability to produce an expansive repertoire of virulence factors, which collectively augment the organism's ability to colonize and invade host tissue, evade host immune responses, and adapt to host-associated environmental challenges. In the laboratory setting, many *S. aureus* cell surface virulence factors are predominantly expressed during exponential phase growth, whereas secreted virulence factors are predominantly expressed as cultures transition to early stationary phase growth [18]. This combined with the recent observation that the modulation of mRNA turnover affects *S. aureus* virulence factor expression led to the premise of the current study; the degradation properties of virulence factors transcripts differ during exponential and stationary phase growth [19], [20], [42]. Further, these changes in mRNA turnover may, in part, account for growth-phase dependent changes in virulence factor expression. A comparison of the mRNA degradation properties of known and predicted virulence factor transcripts indicates that this may be the case; 41% exhibited an alteration in mRNA turnover during exponential in comparison to stationary phase growth. Thus, growth phase-dependent changes in transcript degradation are likely to contribute to changes in the mRNA titers, and consequently protein production, of these virulence determinants.

Our mRNA turnover measurements also revealed growth phase-dependent alterations in mRNA turnover extend beyond virulence factor mRNA species; 884 staphylococcal transcripts are stabilized during stationary phase growth. This phenomenon could be attributable to the stationary phase-dependent down-regulation of components of the organism's RNA degradation machinery. An analysis of the transcript titers of known and putative ribonucleases revealed that neither RNase J1 or RNase Y were differentially expressed in a growth phase dependent manner, whereas RNase HIII and the protein component of RNase P, RnpA, were down-regulated during stationary phase growth.

Bacterial RNase P holoenzymes are ribonucleoprotein complexes consisting of a ribozyme (*rnpB*) and a protein component, RnpA. Both *rnpB* and RnpA are required for bacterial survival, thus an agent that inhibits either subunit's activity could have potential as an antimicrobial therapeutic agent [27], [43]. Indeed, the function of *rnpB* is well established; it catalyzes maturation of precursor tRNA molecules, and progress has been made in developing antimicrobial small molecule inhibitors of *rnpB*-mediated tRNA processing [23], [24], [25]. RnpA augments cellular *rnpB*-precursor tRNA binding [26], a phenotype that is difficult to exploit for antimicrobial screening. In addition to mediating tRNA processing, RNase P has also been shown to catalyze cleavage of other double-stranded RNA substrates, such as guide RNAs, and this activity requires RnpA [30], [31], [32].

Our data suggest that *S. aureus* RnpA exhibits pronounced *in vitro* ribonuclease activity in comparison *S. aureus* RNase J1, RNase Y and RNase HIII, in the conditions assessed here. We do not mean to imply that the other putative ribonucleases assessed are devoid of ribonucleolytic activity. Rather, their activity would probably be better measured in differing buffering conditions or with different RNA substrates. In fact, subsequent studies revealed that *S. aureus* RNase J1 is a potent ribonuclease in differing buffering conditions [10].

By exploiting the observed ribonuclease activity of RnpA, high through-put screening and secondary assays identified small molecule inhibitors of RnpA-mediated *in vitro* RNA degradation. The inhibitory activity of one of these molecules, RNPA1000, was found to have specificity for RnpA, as the agent did not interfere with the activity of commercially available ribonucleases or recombinant *S. aureus* RNase J1 (data not shown). RNPA1000 was shown to have increased antimicrobial activity against RnpA depleted cells, indicating that the agent targets the enzyme *in vivo*. None the less, we cannot exclude the possibility that RNPA1000 may also affect other essential *S. aureus* enzymes. While it remains to be seen whether RnpA's *in vitro* ribonuclease activity correlates to what occurs within the cell, sub-inhibitory concentrations of RNPA1000 and RnpA depleted cells were also found to limit *S. aureus* RNA-degradation, suggesting that RnpA, either alone, as RNase P cofactor, or as a component of a unrecognized complex, may participate in bulk mRNA turnover, a function that may contribute to enzyme's role in maintaining cellular survival. Because *S. aureus* RnpA is well conserved among many Gram-positive bacteria, our findings may have expanded significance by providing insight regarding the RNA degradation machinery of additional bacterial species.

Our results also indicate that inhibitors of RnpA-mediated RNA degradation may have promise as antimicrobial agents. Indeed, RNPA1000 exhibited moderate antimicrobial activity against each of the predominant *S. aureus* MRSA lineages circulating throughout the U.S., VISA, VRSA, VRE, as well as bacterial pathogens with high RnpA amino acid conservation. The agent also limited the organism's pathogenesis in a murine acute lethal model of infection and limited biofilm-associated bacterial burden. While the mode-of-action for RNPA1000 appears to, in part, effect *S. aureus* RnpA mediated RNA processing we do not know if the same is the case for other RNPA1000 susceptible pathogens.

Collectively, these data establish that RnpA represents an attractive antimicrobial drug target and that RNA stabilizing agents represent a new paradigm for treating bacterial pathogens of immense health care concern. The RnpA-inhibitory molecule identified in this study may represent a progenitor of this new class of antimicrobials.

## Methods

### mRNA half-lives

For half life determinations, *S. aureus* strain UAMS-1, RN4220 (pCN51; plasmid containing CdCl_2_ inducible promoter), RN4220 (pRNPA; pCN51 capable of producing full length *rnpA* mRNA), or RN4220 (pRNPA-A.S.; pCN51 capable of producing *rnpA* antisense RNA) were grown to mid-exponential or stationary phase, transcription was arrested by the addition of rifampin (200 µg/ml), and aliquots were removed at 0-, 2.5-, 5-, 15- and 30- min post-transcriptional arrest for strain UAMS-1. To conserve reagents, aliquots were removed at 0 and 10 min post-transcriptional arrest for RN4220 derivatives. Plating ensured cultures had not developed rifampin resistance. Each strain and/or growth phase was assessed twice, except for RN4220 pRNPA-A.S. cells which were assessed four times. RNA was isolated from each aliquot, labeled, hybridized to an *S. aureus* GeneChip (Affymetrix; Santa Clara, CA), duplicates were averaged and the mRNA half-lives of all mRNA species were determined, as previously described [19], [20]. To measure the mRNA turnover characteristics of RNPA1000 challenged cells, exponential-phase *S. aureus* were treated with 0.5X MIC of the RnpA inhibitor or equivalent volume compound solvent (DMSO) for 30 min. Transcript synthesis was then arrested and the transcript titers of mRNA species were measured at 0- and 5- min post-transcriptional arrest [19], [20].

### Protein purification

Each putative *S. aureus* ribonuclease predicted open reading frame was PCR amplified and inserted into the ligation-independent cloning site of plasmid pET-30 Ek/LIC (Novagen; Madison WI). Sequencing confirmed that this fused a hexahistidine-tag to the N-terminus of each protein under the control of the plasmid's isopropyl β-D-1-thiogalactopyranoside (IPTG) inducible promoter. Following transformation, each protein was purified from *E. coli* BL21 (DE3) cells grown in the presence of IPTG (4 hr) by Ni^+2^ affinity chromatography. More specifically, 10 g of cell pellet was suspended in 50 ml of buffer A (300 mM NaCl, 50 mM Na_2_HPO_4_, pH 7.4) containing a complete mini EDTA-free protease inhibitor tablet (Roche; Branford, CT) and 20 mM imidazole. Cells were ruptured by seven passes at 15,000 psi through an Emulsifex-C3 microfluidizer (Avestin Inc.; Ottawa, Canada). Cell debris was removed by centrifugation at 12,000×g for 30 min and supernatants were loaded onto a 5 mL Ni-NTA FF-crude affinity column (GE Healthcare Bio-Sciences; Piscataway, NJ) with an AKTA-FPLC high performance liquid chromatography system (GE Healthcare Bio-Sciences; Pittsburgh, PA). Proteins eluted in a single peak with a linear imidazole gradient (80 mM to 500 mM) in buffer A. The presence of each protein was assessed by Coomassie stained SDS-PAGE and matrix-assisted laser desportion/ionization (MALDI) analysis spectrometry (Wistar Institute; Philadelphia, PA).

### Plasmids

Plasmids pRNPA-S and pRNPA-A.S. contain the putative *rnpA* transcriptional unit including predicted Shine-Dalgarno sequence in the sense and antisense orientation, respectively under control of the CdCl_2_ inducible of the *S. aureus* shuttle-vector pCN51 [38]. Briefly, the *rnpA* open reading frame and 34 nt upstream sequence was PCR amplified from *S. aureus* strain UAMS-1 using primers 5′ GAATTC TCAAATAAAAACGATAAATAAGCGAGTGATGTTA (forward) and 5′ GGTACC TTACTTAATCTTTTTATTAAAAACTTTGGCAA (reverse) containing a 5′ terminal *EcoR*I and *Kpn*I restriction enzyme site (underlined), respectively, or primers in which the restriction enzyme sequence had been reversed. Resulting PCR products were ligated into pCRII-TOPO vector and transformed into *E. coli* INVαF' cells for propagation (Invitrogen, Carlsbad, CA). Plasmid DNA was subsequently purified using QIAprep Spin Miniprep Kits (Qiagen, Valencia, CA) then digested with *EcoR*I *and Kpn*I to liberate the plasmid inserts, which were gel purified using a QIAquick Gel Extraction Kit (Qiagen) and ligated into *EcoR*I *and Kpn*I-digested pCN51. DNA sequencing confirmed the integrity of plasmid pRNPA-S and pRNPA-A.S.

### Western blotting

Affinity purified PolyQuik rabbit *S. aureus* RnpA polyclonal antibodies were generated by Invitrogen (Carlsbad, CA). Total bacterial proteins were isolated from RN4220 cells containing plasmid vector (pCN51), RnpA overexpressor plasmid (pRNPA-S) or RnpA antisense RNA plasmid (pRNPA-A.S.) following 30 min growth in TSB medium supplemented with 2.5 µM CdCl_2_ to induce RNA expression and 10 µg/ml erythromycin for plasmid maintenance. Resultant protein concentrations were determined by conventional Bradford Assays and 2.0 µg of each protein sample or purified *S. aureus* RnpA was electrophoresed in a 10% SDS polyacrylamide gel and transferred to a polyvinylidene fluoride membrane (Millipore, Billerica, MA). Membranes were blocked with 10% milk, washed, incubated with rabbit RnpA antibody (1∶1000 dilution), washed, incubated with horseradish peroxidase-conjugated anti-rabbit antibody (1∶1000 dilution; GE Healthcare) and processed using an Amersham ECL Western Blotting System, according to the manufacturer's recommendations (GE Healthcare).

### RnpA inhibitors

Members of the ActiProbe-25K and Natural Product libraries (29,940 compounds total; TimTec Inc.; Newark, DE) were screened for small molecule inhibitors of *S. aureus* RnpA mediated total bacterial RNA degradation. All reactions (50 µl) were performed in 96-well format and contained 20 pmol RnpA, 200 ng *S. aureus* total RNA, and ∼5 µM of each compound in 1X reaction buffer (2 mM NaCl, 2 mM MgCl_2_, 50 mM Tris-HCl, pH 6.0). Mixtures were incubated at 37°C for 20 min at which time Quant-iT RiboGreen (100 µl; Invitrogen) was added to quantify the amount of RNA substrate remaining. Percent enzyme inhibition was calculated as remaining substrate/starting substrate * 100. For inhibitory titration assays, 1 pmol of *spa* mRNA was incubated with 20 pmol RnpA alone (positive control) or in the presence of increasing amounts (0, 25, 50, 100, 125, 150, 200, 250, and 500 µM) RNP1000 for one hour at 37°C. 20 µl of each reaction mixture were subjected to electrophoresis in a 1.2% formaldehyde-containing agarose gel and visualized by ethidium bromide staining.

### Cytotoxicity assays

HepG2 human hepatocytes (10^5^ cells) were seeded in individual wells of a microtitre plate and incubated for 16 hr at 37°C with 5% carbon dioxide in Dulbecco's Modified Eagle Media supplemented with 10% fetal bovine serum. Cells were then challenged with Mitomycin C (5 µg/ml; positive control) or 0, 25, or 50 µg/ml RNPA1000 for either 24 or 48 hrs. Cell viability was measured spectrophotometrically (570 nm) following the addition and subsequent reduction of tetrazolium salt (MTT) within metabolically active cells, as per the manufacturer's recommendations (American Type Culture Collection; Manassas, VA).

### Antimicrobial susceptibility testing

With the exception of RN4220-derivatives, *in vitro* activities of RNPA1000 against bacteria were determined by the broth microdilution method according to the Clinical and Laboratory Standards Institute (CLSI) guidelines using cation adjusted Mueller-Hinton broth or MH broth supplemented with 5% lysed horse blood (for testing *Streptococcus spp.*). Microtiter plates containing serial dilutions of RNPA1000 (0, 4, 8, 16, 32, 64, and 128 µg/ml) were inoculated with 10^5^ colony forming units (CFU)/ml and incubated for 18 hr at 37°C. The MIC for each isolate was defined as the lowest concentration of RNPA1000 that completely inhibited growth of the organism as detected by the unaided eye. The MIC for each *S. aureus* strain was further refined by repeat testing following the procedure described above, except that microtiter wells contained 1 µg/ml incremental increases in concentration of RNPA1000 spanning the lowest concentration that initially did not completely inhibit growth (16 µg/ml) and the concentration that completely inhibited growth (32 µg/ml). The MIC value for each *S. aureus* strain was determined to be the median score of replicate measurements (*n* = 5). Wells containing concentrations of RNPA1000 ≥ MIC were plated for minimal bactericidal measurement. Where possible, experiments with VRSA strains were performed in a laminar flow hood to minimize potential for equipment contamination. For RN4220 cells containing plasmid vector (pCN51), RnpA overproducing plasmid (pRNPA-S) or RnpA underproducing plasmid (pRNPA-A.S.) *in vitro* antimicrobial activity of RNPA1000 was performed by the microdilution method as described above, except that cells were grown in Tryptic Soy Broth medium supplemented with 2.5 µM CdCl_2_ and 0, 1, 2, 4, 8, 16, 32, 64, or 128 µg/ml RNPA1000. Time-kill assays were also performed to monitor the antimicrobial properties of RNPA1000 for *S. aureus* strain UAMS-1 in the absence and presence of 0.25, 0.5, 2, and 4 times the strain's MIC for oxacillin (1 µg/ml), rifampicin (0.5 µg/ml), vancomycin (2 µg/ml), or daptomycin (1 µg/ml). The indicated amount of RNPA1000 and/or commercial antibiotic were added to mid-exponential phase (2×10^8^ cfu/ml) *S. aureus* strain UAMS-1 cells and incubated at 37°C. Aliquots were removed at 0, 2, 4, 8, and 24 hr post-antimicrobial challenge, serial diluted, and plated to enumerate resulting cfu/ml. All time-kill assays were repeated at least 3 times.

### Biofilm assays

*In vitro* biofilm assays were performed as previously described [40]. Briefly, 1 cm segments of 14-gauge fluorinated ethylene propylene Introcan Safety Catheters (B. Braun, Bethlehem, PA) were coated with human plasma and placed in individual wells of a 12-well microtiter plate containing 2 ml biofilm medium and *S. aureus* strain UAMS-1 at a final OD_600 nm_ of 0.05. Following overnight incubation at 37°C catheters were removed, rinsed in phosphate buffered saline (PBS), and transferred to fresh biofilm medium containing 0, 5, 10, or 20 times the *S. aureus* MIC for RNPA1000. Catheters exposed to each dose (*n* = 3) were recovered daily over a period of 3 days, with the medium being replaced each day. After each recovery time point catheters were rinsed in PBS and adherent bacteria were enumerated by sonication and plating. Analysis of variance (ANOVA) of logarithmically-transformed bacterial count data was used to evaluate the effect of RNPA1000 exposure.

### Acute lethal model of infection

Female 5–6 week old CD-1 mice were challenged by intraperitoneal injection (0.5 ml) of wild type *S. aureus* strain Smith, resulting in a final inoculum of 4.55×10^5^ colony forming units/animal; equivalent to 10–100 LD_50_s and resulted in death of non-treated control animals (*n* = 5) within 24 hr post-inoculum. RNPA1000 was solubilized in 1∶1 mixture of DMSO and PEG400; Vancomycin was prepared in water. Animals (5/dose group) were administered 16, 64, and 256 mg/kg or 0.25, 1, 4, and 16 mg/kg of RNPA1000 or Vancomycin, respectively, at 30 min post infection by subcutaneous injection (0.2 ml). The percent surviving animals receiving no treatment, a single dose of Vancomycin, or RnpA-inhibitor was recorded daily over the course of the study (5 days).

### Ethics statement

All animal studies were performed at the University of North Texas Health Science Center (UNTHSC) at Fort Worth under the principles and guidelines described in the Guide for the Care and Use of Laboratory Animals. UNTHSC is an American Association for Laboratory Animal Science (AALAS) and United States Department of Agriculture (USDA) accredited facility using Institutional Animal Care and Use Committee (IACUC) approved protocol UNT 2006/07-09. The UNTHSC Animal Program is registered with the Office of Laboratory Animal Welfare (OLAW Animal Welfare Assurance No. A37711-01).

## Supporting Information

Figure S1 RnpA expression. (A) Plotted are the growth characteristics (optical density; Y-axis), for *S. aureus* strain RN4220 containing vector (pCN51; dark blue diamonds), *rnpA* sense RNA (pRNPA-S; dark blue triangles) and *rnpA* antisense RNA (pRNPA-A.S.; red squares) when grown in the presence of 10 µM CdCl2. Plasmid capable of producing an RNA complementry to rnpA mRNA exhibited diminished growth for a period of 4 hrs (X-axis) in the presence of inducer. This growth defect was not observed when cells were grown in the absence of cadmium chloride (not shown) or when grown in the presence of 2.5 µM CdCl2 (hashed line and pink squares). (B) Western blotting results for *S. aureus* strain RN4220 pCN51 (vector), RN4220 pRNPA (overexpressor), and RN4220 pRNPA-A.S. (RnpA depleted) cells grown in the presence of 2.5 µM CdCl2.
(0.29 MB TIF)

Figure S2 *S. aureus* time-kill assay results. (A) Mid-exponential phase *S. aureus* strain UAMS-1 cells were treated with 0.25, 0.5, 1, 2, or 4 times the MIC for RNPA1000. Plotted are the average cfu/ml at 0, 2, 4, 8, and 24 hr post-RNPA1000 addition for each drug concentration tested (*n* = 3); standard deviation shown. (B) Plotted are the average cfu/ml at 2, 4, 8, and 24 hr post-oxacillin treatment (0.25, 0.5, 2, or 4 times the MIC; *n* = 3) of mid-exponential phase cells. (C) Mid-exponential phase cells were treated with 0.5 times the MIC for RNPA1000, oxacillin, or both (RNPA1000 and oxacillin). Shown are the average cfu/ml of mid exponential phase cells following 2, 4, 8, and 24 hr post treatment (*n* = 3); standard deviation shown.
(0.30 MB TIF)

Figure S3 RnpA amino acid comparisons. Alignment of amino acid sequences of RnpA using GramAlign (http://bioinfo.unl.edu/gramalign.php) with default parameters. Conserved amino acids are boxed.
(2.93 MB TIF)

Table S1 mRNA half-lives of S. aureus transcripts producedduring exponential or stationary phase growth. Shown are the expression properties and RNA half-lives of all S. aureus transcripts produced during exponential and/or stationary phase growth.
(3.46 MB XLS)

Table S2 Characterization of stationary phase-specific small stable RNAs. Characterization of Stationary Phase-Specific Small Stable RNAs.
(0.03 MB XLS)

Table S3 In vivo efficiacy of RnpA-inhibitor. Acute-lethal murine model of infection results of animals treated with the RnpA-inhibitor RNPA1000, Vancomycin (positive control) and mock treatment (negative control).
(0.02 MB XLS)

## References

[1] Shorr AF, Tabak YP, Killian AD, Gupta V, Liu LZ (2006). Healthcare-associated bloodstream infection: A distinct entity? Insights from a large U.S. database.. Crit Care Med.

[2] Bancroft EA (2007). Antimicrobial resistance: it's not just for hospitals.. Jama.

[3] Klevens RM, Morrison MA, Nadle J, Petit S, Gershman K (2007). Invasive methicillin-resistant *Staphylococcus aureus* infections in the United States.. Jama.

[4] Appelbaum PC (2007). Reduced glycopeptide susceptibility in methicillin-resistant *Staphylococcus aureus* (MRSA).. Int J Antimicrob Agents.

[5] Zetola N, Francis JS, Nuermberger EL, Bishai WR (2005). Community-acquired meticillin-resistant *Staphylococcus aureus*: an emerging threat.. Lancet Infect Dis.

[6] Carpousis AJ (2007). The RNA degradosome of *Escherichia coli*: an mRNA-degrading machine assembled on RNase E.. Annu Rev Microbiol.

[7] Mackie GA (1998). Ribonuclease E is a 5′-end-dependent endonuclease.. Nature.

[8] Vanzo NF, Li YS, Py B, Blum E, Higgins CF (1998). Ribonuclease E organizes the protein interactions in the *Escherichia coli* RNA degradosome.. Genes Dev.

[9] Condon C (2003). RNA processing and degradation in *Bacillus subtilis*.. Microbiol Mol Biol Rev.

[10] Even S, Pellegrini O, Zig L, Labas V, Vinh J (2005). Ribonucleases J1 and J2: two novel endoribonucleases in *B. subtilis* with functional homology to *E. coli* RNase E.. Nucleic Acids Res.

[11] Mathy N, Benard L, Pellegrini O, Daou R, Wen T (2007). 5′-to-3′ exoribonuclease activity in bacteria: role of RNase J1 in rRNA maturation and 5′ stability of mRNA.. Cell.

[12] Commichau FM, Rothe FM, Herzberg C, Wagner E, Hellwig D (2009). Novel activities of glycolytic enzymes in *Bacillus subtilis*: interactions with essential proteins involved in mRNA processing.. Mol Cell Proteomics.

[13] Mader U, Zig L, Kretschmer J, Homuth G, Putzer H (2008). mRNA processing by RNases J1 and J2 affects *Bacillus subtilis* gene expression on a global scale.. Mol Microbiol.

[14] Yao S, Sharp JS, Bechhofer DH (2009). *Bacillus subtilis* RNase J1 endonuclease and 5′ exonuclease activities in the turnover of DeltaermC mRNA.. Rna.

[15] Shahbabian K, Jamalli A, Zig L, Putzer H (2009). RNase Y, a novel endoribonuclease, initiates riboswitch turnover in *Bacillus subtilis*.. Embo J.

[16] Lehnik-Habrink M, Pfortner H, Rempeters L, Pietack N, Herzberg C (2010). The RNA degradosome in *Bacillus subtilis*: identification of CshA as the major RNA helicase in the multiprotein complex.. Mol Microbiol.

[17] Kobayashi K, Ehrlich SD, Albertini A, Amati G, Andersen KK (2003). Essential *Bacillus subtilis* genes.. Proc Natl Acad Sci U S A.

[18] Novick RP (2003). Autoinduction and signal transduction in the regulation of staphylococcal virulence.. Mol Microbiol.

[19] Anderson KL, Roberts C, Disz T, Vonstein V, Hwang K (2006). Characterization of the *Staphylococcus aureus* heat-shock, cold-shock, stringent, and SOS responses and their effects on log-phase mRNA turnover.. J Bacteriol.

[20] Roberts C, Anderson KL, Murphy E, Projan SJ, Mounts W (2006). Characterizing the Effect of the *Staphylococcus aureus* Virulence Factor Regulator, SarA, on Log-Phase mRNA Half-Lives.. J Bacteriol.

[21] Takayama K, Kjelleberg S (2000). The role of RNA stability during bacterial stress responses and starvation.. Environ Microbiol.

[22] Anderson KL, Dunman PM (2009). Messenger RNA Turnover Processes in *Escherichia coli*, *Bacillus subtilis*, and Emerging Studies in *Staphylococcus aureus*.. Int J Microbiol.

[23] Frank DN, Pace NR (1998). Ribonuclease P: unity and diversity in a tRNA processing ribozyme.. Annu Rev Biochem.

[24] Kazantsev AV, Pace NR (2006). Bacterial RNase P: a new view of an ancient enzyme.. Nat Rev Microbiol.

[25] Walker SC, Engelke DR (2006). Ribonuclease P: the evolution of an ancient RNA enzyme.. Crit Rev Biochem Mol Biol.

[26] Hartmann RK, Gossringer M, Spath B, Fischer S, Marchfelder A (2009). The making of tRNAs and more - RNase P and tRNase Z.. Prog Mol Biol Transl Sci.

[27] Gossringer M, Kretschmer-Kazemi Far R, Hartmann RK (2006). Analysis of RNase P protein (*rnpA*) expression in *Bacillus subtilis* utilizing strains with suppressible rnpA expression.. J Bacteriol.

[28] Schedl P, Primakoff P (1973). Mutants of *Escherichia coli* thermosensitive for the synthesis of transfer RNA.. Proc Natl Acad Sci U S A.

[29] Waugh DS, Pace NR (1990). Complementation of an RNase P RNA (*rnpB*) gene deletion in *Escherichia coli* by homologous genes from distantly related eubacteria.. J Bacteriol.

[30] Lundblad EW, Xiao G, Ko JH, Altman S (2008). Rapid selection of accessible and cleavable sites in RNA by *Escherichia coli* RNase P and random external guide sequences.. Proc Natl Acad Sci U S A.

[31] Liu F, Altman S (1994). Differential evolution of substrates for an RNA enzyme in the presence and absence of its protein cofactor.. Cell.

[32] Marvin MC, Engelke DR (2009). Broadening the mission of an RNA enzyme.. J Cell Biochem.

[33] Letunic I, Copley RR, Pils B, Pinkert S, Schultz J (2006). SMART 5: domains in the context of genomes and networks.. Nucleic Acids Res.

[34] Schultz J, Milpetz F, Bork P, Ponting CP (1998). SMART, a simple modular architecture research tool: identification of signaling domains.. Proc Natl Acad Sci U S A.

[35] Spitzfaden C, Nicholson N, Jones JJ, Guth S, Lehr R (2000). The structure of ribonuclease P protein from *Staphylococcus aureus* reveals a unique binding site for single-stranded RNA.. J Mol Biol.

[36] Chaudhuri RR, Allen AG, Owen PJ, Shalom G, Stone K (2009). Comprehensive identification of essential *Staphylococcus aureus* genes using Transposon-Mediated Differential Hybridisation (TMDH).. BMC Genomics.

[37] Ji Y, Zhang B, Van SF, Horn, Warren P (2001). Identification of critical staphylococcal genes using conditional phenotypes generated by antisense RNA.. Science.

[38] Charpentier E, Anton AI, Barry P, Alfonso B, Fang Y (2004). Novel cassette-based shuttle vector system for gram-positive bacteria.. Appl Environ Microbiol.

[39] McDougal LK, Steward CD, Killgore GE, Chaitram JM, McAllister SK (2003). Pulsed-field gel electrophoresis typing of oxacillin-resistant *Staphylococcus aureus* isolates from the United States: establishing a national database.. J Clin Microbiol.

[40] Weiss EC, Spencer HJ, Daily SJ, Weiss BD, Smeltzer MS (2009). Impact of *sarA* on antibiotic susceptibility of *Staphylococcus aureus* in a catheter-associated in vitro model of biofilm formation.. Antimicrob Agents Chemother.

[41] Beenken KE, Dunman PM, McAleese F, Macapagal D, Murphy E (2004). Global Gene Expression in *Staphylococcus aureus* Biofilms.. J Bacteriol.

[42] Huntzinger E, Boisset S, Saveanu C, Benito Y, Geissmann T (2005). *Staphylococcus aureus* RNAIII and the endoribonuclease III coordinately regulate spa gene expression.. EMBO J.

[43] Kole R, Baer MF, Stark BC, Altman S (1980). *E. coli* RNAase P has a required RNA component.. Cell.

