# Supplementary figures and images for: Small Molecule Inhibitors of Staphylococcus aureus RnpA Alter Cellular mRNA Turnover, Exhibit Antimicrobial Activity, and Attenuate Pathogenesis
Source: PLoS Pathog. 2011 Feb 10;7(2):e1001287. doi: 10.1371/journal.ppat.1001287 (PMC3037362; doi:10.1371/journal.ppat.1001287)

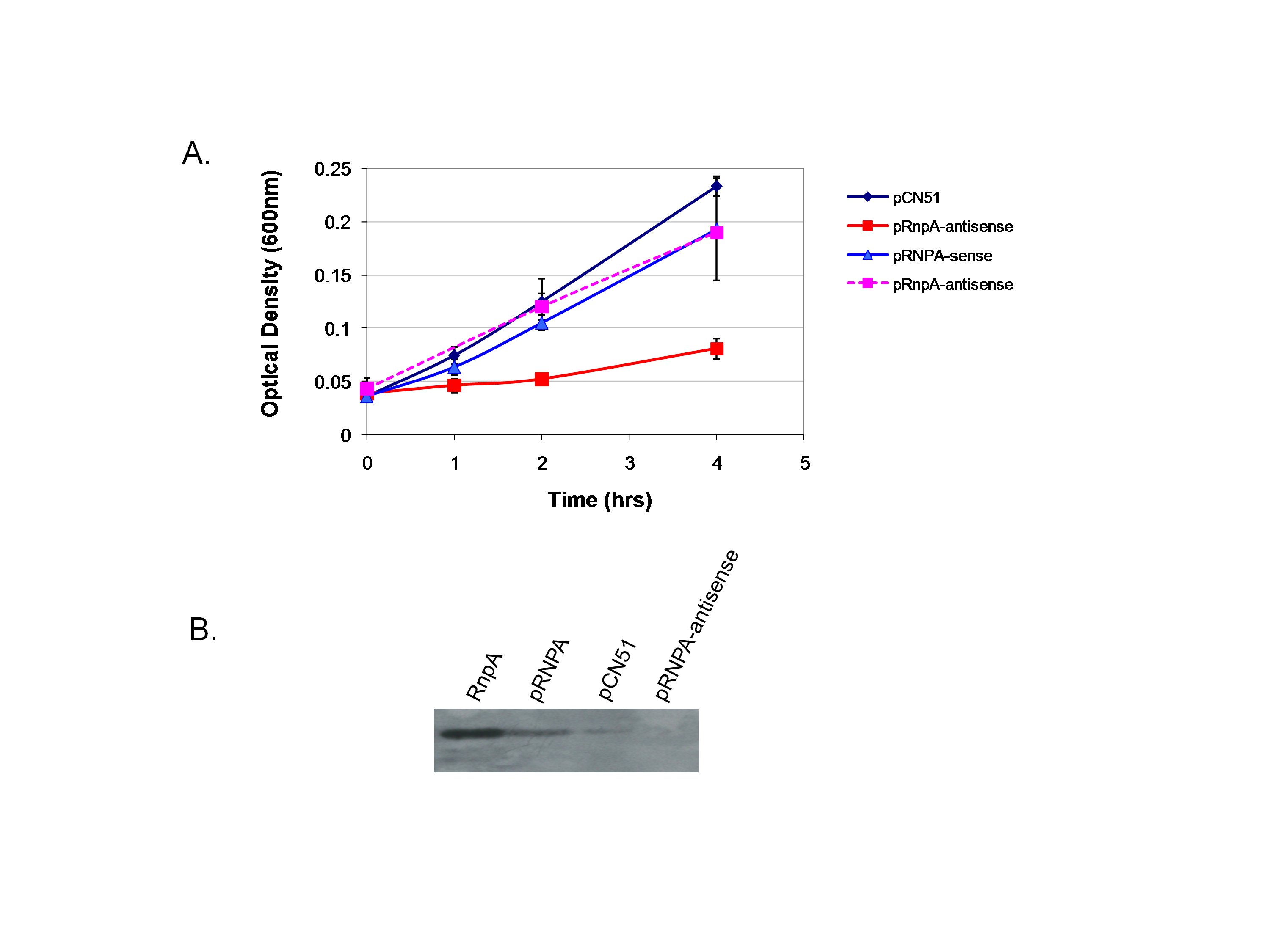

Supplement: Figure S1 — RnpA expression. (A) Plotted are the growth characteristics (optical density; Y-axis), for S. aureus strain RN4220 containing vector (pCN51; dark blue diamonds), rnpA sense RNA (pRNPA-S; dark blue triangles) and rnpA antisense RNA (pRNPA-A.S.; red squares) when grown in the presence of 10 µM CdCl2. Plasmid capable of producing an RNA complementry to rnpA mRNA exhibited diminished growth for a period of 4 hrs (X-axis) in the presence of inducer. This growth defect was not observed when cells were grown in the absence of cadmium chloride (not shown) or when grown in the presence of 2.5 µM CdCl2 (hashed line and pink squares). (B) Western blotting results for S. aureus strain RN4220 pCN51 (vector), RN4220 pRNPA (overexpressor), and RN4220 pRNPA-A.S. (RnpA depleted) cells grown in the presence of 2.5 µM CdCl2. (0.29 MB TIF) [file ppat.1001287.s001.tif]

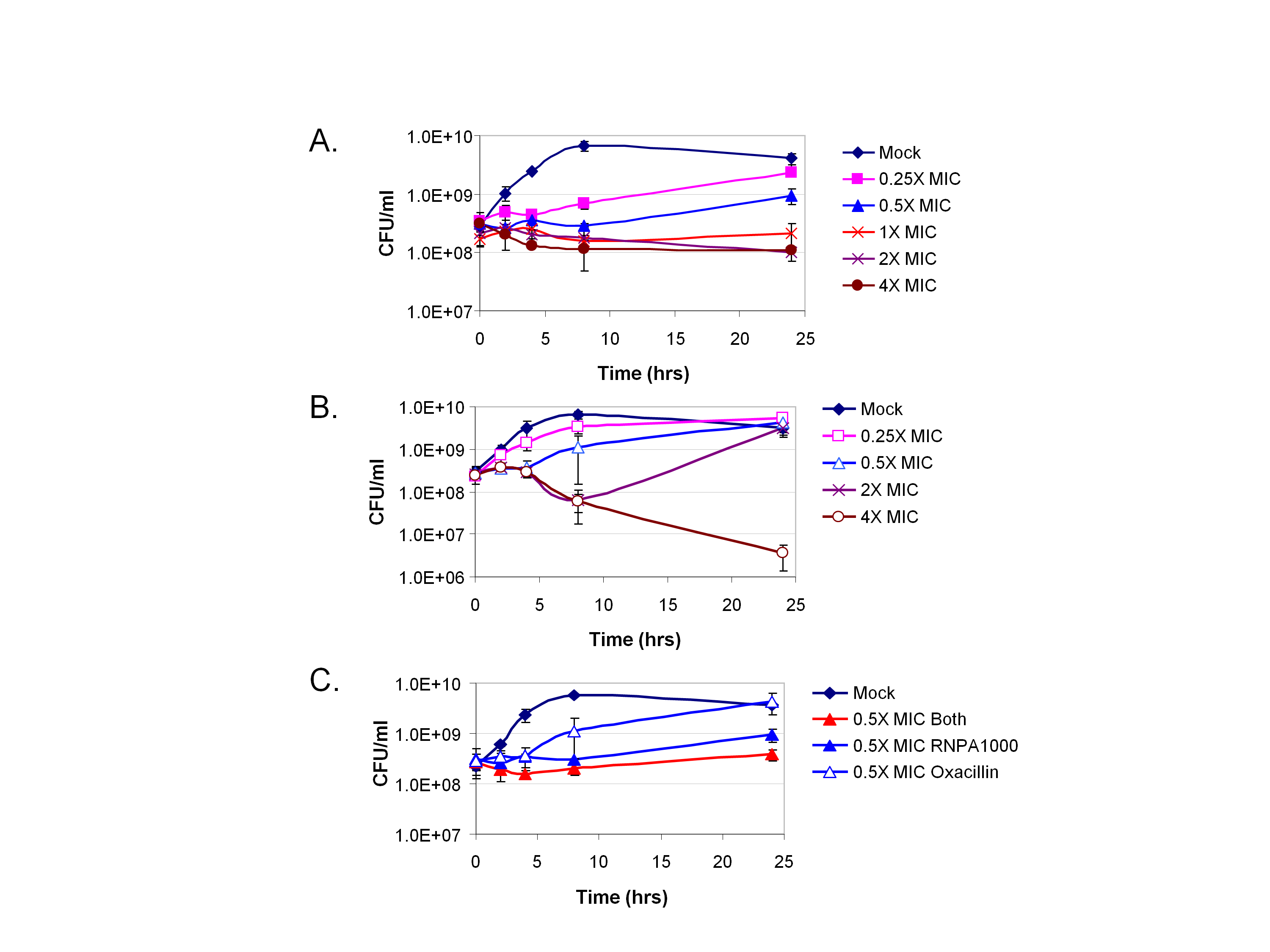

Supplement: Figure S2 — S. aureus time-kill assay results. (A) Mid-exponential phase S. aureus strain UAMS-1 cells were treated with 0.25, 0.5, 1, 2, or 4 times the MIC for RNPA1000. Plotted are the average cfu/ml at 0, 2, 4, 8, and 24 hr post-RNPA1000 addition for each drug concentration tested (n = 3); standard deviation shown. (B) Plotted are the average cfu/ml at 2, 4, 8, and 24 hr post-oxacillin treatment (0.25, 0.5, 2, or 4 times the MIC; n = 3) of mid-exponential phase cells. (C) Mid-exponential phase cells were treated with 0.5 times the MIC for RNPA1000, oxacillin, or both (RNPA1000 and oxacillin). Shown are the average cfu/ml of mid exponential phase cells following 2, 4, 8, and 24 hr post treatment (n = 3); standard deviation shown. (0.30 MB TIF) [file ppat.1001287.s002.tif]

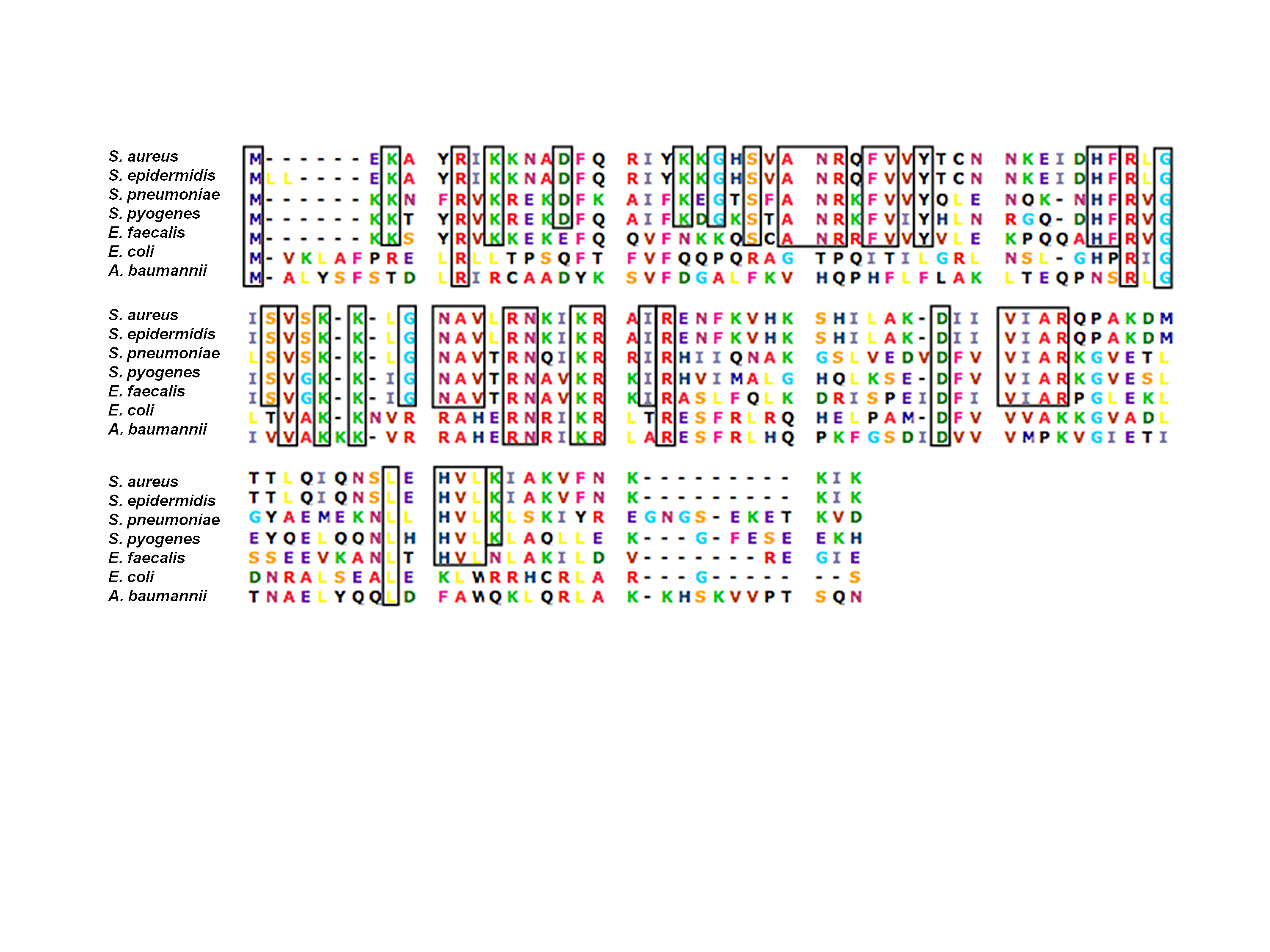

Supplement: Figure S3 — RnpA amino acid comparisons. Alignment of amino acid sequences of RnpA using GramAlign (http://bioinfo.unl.edu/gramalign.php) with default parameters. Conserved amino acids are boxed. (2.93 MB TIF) [file ppat.1001287.s003.tif]
